# Diagnostics différentiels d'images d'hypofixations sur une scintigraphie du squelette: à propos d'un cas de leucémie aigue lymphoblastique

**DOI:** 10.11604/pamj.2016.24.146.8328

**Published:** 2016-06-15

**Authors:** Nisrine Bahadi, Abdelhamid Biyi, Salah Nabih Oueriagli, Abderrahim Doudouh

**Affiliations:** 1 Service de Médecine Interne, Hôpital Militaire d'Instruction Mohammed V de Rabat, Université Mohammed V de Rabat, Maroc; 2 Service de Médecine Nucléaire, Hôpital Militaire d'Instruction Mohammed V de Rabat, Université Mohammed V de Rabat, Maroc

**Keywords:** Scintigraphie osseuse, hypofixation, leucémie aigue lymphoblastique, Bone scan, reduced uptake, acute lymphoblastic leukemia

## Abstract

Si les hyperfixations sont la traduction scintigraphique habituelle de nombreuses pathologies osseuses, les hypofixations sont un événement rare et suscitent souvent analyse minutieuse afin d’éviter des interprétations erronés. Nous rapportons ici l'observation d'un adolescent de 17 ans admis pour douleurs osseuses diffuses, une hypercalcémie et une thrombopénie. La scintigraphie du squelette a montré des foyers d'hypofixations. L’étude de la moelle osseuse a conclu au diagnostic de leucémie aigue lymphoblastique. A travers ce cas clinique, nous discutons les principaux diagnostics différentiels soulevés par de telles anomalies scintigraphiques.

## Abstract (en)

If increased uptake during bone scan usually bring to light many bone pathologies, reduced uptakes are a rare occurrence and they require careful analysis to avoid erroneous interpretations. We report the case of a 17-year old admitted with diffuse bone pain, hypercalcemia and thrombopenia. Bone scan showed areas of low uptakes. Bone marrow tests allowed the diagnosis of acute lymphoblastic leukemia. This case report aims to discuss the main differential diagnoses based on such bone scan abnormalities.

## Introduction

Contrairement aux tumeurs solides, il est rare voir une scintigraphie osseuse s'inscrire dans le bilan initial ou bien du suivi d'hémopathies malignes. Dans les leucémies aigues en particulier, elle est souvent demandée avant le diagnostic pour étiqueter des douleurs osseuses inexpliquées, un trouble du métabolisme phosphocalcique, ou bien une élévation des marqueurs du remodelage osseux. Les anomalies scintigraphiques sont loin d’être spécifiques d'où la difficulté de la recherche étiologique. Dans ce qui suit, nous rapportons l'observation d'un adolescent atteint de leucémie aigue lymphoblastique révélée par un tableau algique inaugural sévère. La scintigraphie du squelette avait alors révélé des images d'ostéonécrose. Les principaux diagnostics différentiels soulevés par de telles anomalies scintigraphiques seront discutés.

## Patient et observation

Il s'agissait d'un patient âgé de 17 ans, sans antécédent notable, qui avait été admis aux urgences devant l'installation brutale de douleurs abdominales sans trouble du transit mais accompagnées de fièvre à 39°C, d'un syndrome inflammatoire biologique (CRP à 104 mg/l), d'hypercalcémie à 130mg/L, de thrombopénie à 30 000 plaquette par ml, sans syndrome hémorragique ni anémique. La phosphorémie, la kaliémie et l'amylasémie étaient normales. Le dosage des D-Dimers était négatif et le taux de fibrinogène était également normal. L’échographie abdominale et la TDM thoraco-abdomino-pelvienne ne retrouvaient pas de syndrome tumoral notamment pas d'adénopathies profondes ni de splénomégalie. L’état du patient s’était amélioré sous traitement symptomatique comprenant hyper hydratation et antalgiques. Le reste du bilan étiologique était négatif. En effet, les radiographies du squelette étaient normales. Le taux de parathormone 1-84 était plutôt bas (2 pg/ml VN: 15 à 65 pg/ml). Le myélogramme montrait une moelle riche réactionnelle sans autre anomalie des trois lignées et sans images d'hémophagocytose médullaire.

**Figure 1:**
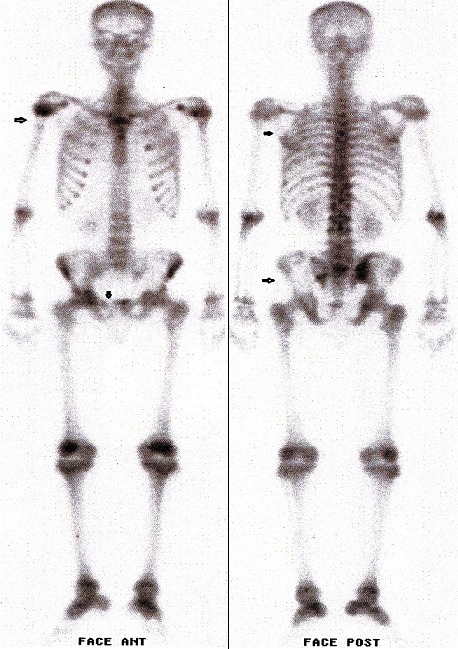
Scintigraphie du squelette en face antérieure et postérieure montrant des foyers d'hypofixation sur le bord externe de l'omoplate gauche, le tiers supérieur de l'humérus droit, la branche iliopubienne droite, l'aile iliaque gauche et l'articulation sacro-iliaque du même coté

**Figure 2:**
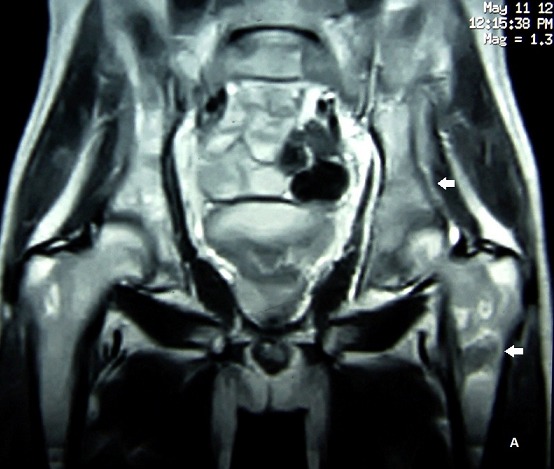
IRM du bassin et des fémurs (coupe frontale) montrant des foyers d'hypersignal sur les coupes pondérées en séquences T2

Trois semaines plus tard, le patient est revenu avec la même symptomatologie clinique bruyante, toujours une hypercalcémie, des douleurs osseuses diffuses, et cette fois, une bicytopénie (thrombopénie et anémie normochrome normocytaire). Le fer sérique était normal, mais le taux de ferritine à 1094 ng/ml (VN: 11 à 336 ng/ml). La recherche de schizocytes sur le frottis sanguin était négative. La scintigraphie osseuse réalisée 3H après injection intraveineuse de 550 MBq de 99mTc-HMDP montrait des plages d'hypofixation sur le bord externe de l'omoplate gauche, le tiers supérieur de l'humérus droit, la branche iliopubienne droite et l'aile iliaque gauche s’étendant jusqu’à l'articulation sacro-iliaque du même coté. Aucune autre anomalie n'a été décelée sur le reste du squelette (Figure 1). Les phosphatases alcalines étaient normales, et fait troublant vu l’âge du patient, l'acide urique était à 101 mg/l (VN: 36 à 70 mg/ml) sans anomalie de la fonction rénale. L'IRM du rachis et du bassin montrait de multiples anomalies du signal des corps vertébraux, des ailes iliaques et du sacrum, tantôt sous forme d'hypersignal tantôt sous forme d'hyposignal sur les coupes pondérées en séquences T1 et T2 (Figure 2). Ces anomalies étaient en faveur d'ostéonécroses à différents stades d’évolution allant par endroits jusqu’à la fibrose. Un dosage de la B-glucosidase acide a été demandé et montrait un taux normal de cette activité enzymatique. L’électrophorèse de l'hémoglobine l’était également normale. Devant la persistance de la symptomatologie douloureuse nous avions décidé de refaire un myélogramme qui était cette fois en faveur d'une leucémie aigue lymphoblastique. Le caryotype n'avait pas été fait. Le patient est malheureusement décédé dans le mois qui a suivi le diagnostic après un épisode d'hémorragique fait d’épistaxis et d'hématémèses de grande abondance.

## Discussion

En raison de l’âge du patient (17 ans), les tableaux clinique et biologique et la constatation de foyers d'hypofixations sur le squelette nous ont fait penser d'abord à une maladie de Gaucher. C'est une maladie grave chez l'enfant et l'adolescent mais actuellement curable dans certaines formes [1]. Elle est due à une surcharge lysosomale en glucocérébroside et se transmet selon le mode autosomique récessif. Son diagnostic repose sur le dosage de l'activité de la glucocérébrosidase dans les leucocytes. Anémie et thrombopénie sont fréquentes en cas de maladie de Gaucher et peuvent être expliquées par l'hypersplénisme. Notre patient n'avait pas de splénomégalie. Par ailleurs, les hypofixations migrent sur les examens scintigraphiques successifs et suivent la topographie de la douleur dans la maladie de Gaucher [2]. Enfin le dosage de l'activité de la glucocérébrosidase chez notre patient était négatif. La drépanocytose peut également être responsable d'infarctus osseux et d'une élévation du taux de ferritine sérique. La scintigraphie aux radio-biphosphonates peut montrer des images d'hypofixation quand les clichés sont réalisés précocement [3]. Plus tard, ces hypofixations laissent la place à des hyperfixations ou bien des images mixtes. On peut également observer une fixation de la rate [4] en rapport avec des infarctus spléniques. Le diagnostic de drépanocytose reste biologique et repose sur l’électrophorèse de l'hémoglobine. La recherche d'hémoglobine fœtale s'est révélée négative chez notre patient. Des foyers d'hypofixation sur le squelette peuvent également être secondaires à une coagulation intra vasculaire disséminée [5], mais là encore et malgré la thrombopénie, le dosage du fibrinogène et du D-Dimère qui est un produit de dégradation de la fibrine a permit d’écarter ce diagnostic. La thrombopénie initiale avait fait discuter l’éventualité d'une microangiopathie thrombotique d'autant plus qu'elle était accompagnée d'une anémie à la seconde admission du patient. Cependant, l'absence de schizocytes sur le frottis sanguin nous a fait renoncer à cette piste.

Les leucémies représentent 30 à 40% des cancers chez l'enfant. La forme aigue lymphoblastique est la plus fréquente et représente 85% des cas. Les manifestations ostéoarticulaires sont inaugurales dans un tiers des LAL [6]. Les ostéonécroses, habituellement rattachées à une origine iatrogène, sont souvent source de morbidité chez les enfants atteints de leucémies aigues, même après rémission. Leur diagnostic précoce est donc d'une importance capitale, avant l'installation des complications ostéoarticulaires. Dans cette optique, il est utile de souligner l'apport de l'IRM du corps entier [7]. Elle permet de faire une cartographie des lésions qui peuvent être asymptomatiques dans environ 55% des cas, et peut modifier, par la topographie des atteintes et leur sévérité, le protocole thérapeutique (changement de corticoïde, utilisation de biphosphonates). La scintigraphie osseuse quant à elle s'inscrit rarement dans le cadre du bilan d'une leucémie aigue. Dans la plus-part des cas, elle est pratiquée avant le diagnostic, devant une symptomatologie douloureuse bruyante et inexpliquée. Chez notre patient, elle avait était demandée pour expliquer les douleurs osseuses et l'hypercalcémie. Cette dernière serait la conséquence d'une stimulation de la résorption osseuse en rapport avec une sécrétion de parathyroid hormone related protein par les cellules leucémiques [8]. L'hypercalcémie s'accompagne d'anomalies de fixation osseuse et/ou extra-osseuse très largement documentées [4, 9] mais absentes dans le cas présent. La topographie des anomalies scintigraphiques observées au cours des leucémies aigues est très variable et dépend de l’âge des patients. Chez les enfants, Shalaby-Rana & al [10] et Clausen & al [11] rapportent une nette prédilection des atteintes métaphyso-diaphysaires des os long, en particuliers des membres inférieurs. Les atteintes costales sont plutôt fréquentes chez adultes [12]. Les images d'hypofixation sont plus rares et ont été rapportée dans des observations isolées [13].

Des hypofixations vertébrales et des tassements ont également étaient signalés. Par ailleurs, Shalaby-Rana et al insistent sur l'intérêt des acquisitions précoces du corps entier, qui permettent de mettre en évidence plus d'anomalies que les acquisitions tardives. Des hyperfixations diffuses des os du bassin et du rachis peuvent être observées uniquement aux temps précoces de l'examen et signifieraient une infiltration de la moelle osseuse [10]. Dans tous les cas, ces atteintes osseuses ne modifient heureusement pas le pronostic des leucémies aigues chez l'enfant [14]. Il est enfin utile de rappeler que des hypofixations sur une scintigraphie du squelette peuvent être en rapport avec certaines tumeurs bénignes (hémangiome [15]), des métastases (thyroïde, rein, neuroblastome [5, 16, 17]), certains traitements anticancéreux notamment la radiothérapie et le traitement par les ultrasons (hypofixations costales chez des patients atteints hépatocarcinomes [18]), une infection osseuse [5], ou bien rester dans de rares cas sans cause décelable [19].

## Conclusion

Bien que rares, les hypofixations sur une scintigraphie du squelette suscitent beaucoup d'interrogations. L'absence de spécificité suppose une bonne connaissance des éventualités diagnostiques pour une meilleure orientation des explorations supplémentaires. Chez l'enfant et l'adolescent, et étant donnée la gravité de cette maladie et la possibilité d'un traitement curatif, des douleurs osseuses inexpliquées devraient inciter à pratiquer une scintigraphie du squelette combinant de préférence une exploration du corps entier aux temps précoce et tardif.
